# Stress-Activated Protein Kinase Pathways as Potential Targets for the Development of New Antifungals

**DOI:** 10.3390/jof12020142

**Published:** 2026-02-14

**Authors:** Rebeca Alonso-Monge, José Pedro Guirao-Abad, Juan Carlos Argüelles

**Affiliations:** 1 Unidad de Microbiología, Departamento de Microbiología y Parasitología, Facultad de Farmacia, Universidad Complutense de Madrid, Plaza de Ramón y Cajal s/n, E-28040 Madrid, Spain; 2 Division of Basic and Translational Science, Department of Emergency Medicine, The Ohio State University Wexner Medical Center, Colombus, OH 43210, USA; jose.guiraoabad@osumc.edu; 3 Área de Microbiología, Facultad de Biología, Universidad de Murcia, E-30071 Murcia, Spain; arguelle@um.es

**Keywords:** antifungals, high-priority fungal pathogens, stress-activated kinases (SAPK), HOG pathway

## Abstract

The World Health Organization WHO considers fungal infections as a significant global risk that necessitates the development of new therapies. The arsenal of antifungals is limited, and the eukaryotic organization of fungi makes it difficult to find selective antifungal targets. In the search for potential targets for the design of new antifungals, the Stress-Activated Protein Kinase (SAPKs) pathways, and specifically, the two-component system, could be a plausible option since this upstream signaling system is absent in metazoans. SAPK pathways are involved in the response and adaptation to different environmental conditions. In pathogenic fungi, these signaling pathways are crucial for virulence, and some of them become activated in response to certain antifungals. Although further experimental evidence is required on the role of SAPKs in antifungal signaling and resistance, the possibility of impairing SAPK signaling by tagging the two-component system can be considered a useful strategy for implementing future antifungal therapies. In particular, the beneficial value of SAPK modulators combined with antifungal drugs should be a preferred line of research. In this review, we focused on the connection between the SAPK pathways and antifungal signaling in the four fungal priority pathogens, *Cryptococcus neoformans*, *Candidozyma* (formerly *Candida*) *auris*, *Aspergillus fumigatus*, and *Candida albicans*, defined by the WHO.

## 1. Introduction

In the last few decades, fungal infections have undergone a dramatic rise worldwide, being responsible for approximately 1.7 million deaths per year [1], although another study increases this number to 2.5 million [2]. In 2022, the World Health Organization (WHO) published the first list of fungal priority pathogens, with the intention to guide new research, development, and public health actions [3]. This list categorizes fungal infections into three priority groups: critical, high, and medium. The critical group includes *Cryptococcus neoformans*, *Candidozyma* (formerly *Candida*) *auris*, *Aspergillus fumigatus*, and *Candida albicans*.

Fungi have traditionally been considered less significant pathogens than bacteria or viruses, and, therefore, they have received limited attention from both public organizations and pharmaceutical companies [4]. However, the number of deaths attributable to the main invasive fungal diseases is comparable to those produced by tuberculosis or malaria [5]. This scenario has worsened in recent decades due to the increasing isolation of fungal pathogens responsible for outbreaks in both hospitals and community settings, mainly associated with the increase in the immunocompromised population. Furthermore, the opportunistic pan-resistant yeast *Candidozyma* (*Candida*) *auris* represents a serious concern in intensive care units and among the immunodepleted people [1,6,7]. In addition, the growing number of fungal strains resistant to conventional antifungal compounds complicates the development of a safe and effective chemotherapy against highly prevalent infectious fungi [8,9,10].

Another problem comes from the worrying rise in resistant strains [11]. The mechanisms involved in resistance are complex [12] and imply secondary pathways that contribute to the whole toxicity. These new routes are frequently poorly understood [13]. Excluding certain molecules applied to specific mycosis, the three main families of antifungal drugs currently in use (Polyenes, Azoles, and Echinocandins) are classified according to their well-established chemical structure and mechanism of action, so far established, rather than their host range. Given the availability of several comprehensive revisions on this topic, we will provide brief comments on some relevant aspects that have not been sufficiently addressed previously [4,14,15,16].

### 1.1. Polyenes

The polyene amphotericin B (AMB) remains one of the most widely used compounds in clinical therapy to treat most superficial and severe fungal infections, such as cryptococcosis, candidiasis, aspergillosis, histoplasmosis, and coccidioidomycosis [15]. Polyenes specifically bind to the ergosterol present in the fungal plasma membrane, causing a loss of permeability and metabolic alterations in the host. Additionally, AMB induces the production of Reactive Oxygen Species (ROS), contributing to its fungicidal effect [17]. The therapeutic value of this polyene is reinforced by the fact that resistance to AMB remains unexpectedly rare after long daily application in clinical therapy [18]. Furthermore, its undesirable side effects, like nephro- and hepatotoxicity as well as allergic reactions, have been surmounted by novel liposomal formulations and other innovative drug formulations, which are less toxic but have increased the economic costs of their prescription [4]. In contrast, nystatin is less commonly used because of its higher toxicity and poor absorption through the digestive tract. The pleiotropic mechanism of action of polyenes has been extensively analyzed in other works [19,20,21,22].

### 1.2. Azoles

This group of cyclic organic molecules contains the largest number of antifungals. In particular, the former triazoles (i.e., fluconazole (FLC) and itraconazole (ITC), and those of the second generation (i.e., voriconazole (VRC) or posaconazole (PSZ)) show high efficacy against opportunistic mycosis, and are applied for prophylaxis of invasive infections caused by *Candida* spp. Azoles are also used to treat aspergillosis caused mainly by *A. fumigatus* [11,23], although VRC and ITC-resistant strains of *A. fumigatus* have been isolated in different European countries [24]. Azoles inhibit the fungal cytochrome P450-dependent enzyme lanosterol 14-α-demethylase, causing an ergosterol deficit, which impairs the correct structure of the cell membrane and accumulation of toxic sterol precursors. One of the main drawbacks is that azoles act essentially as fungistatic rather than fungicide drugs. For a better understanding of their mechanism of action and the disadvantages of administering them, the following documents are available [15,16]. A matter of concern stems from the fact that resistance to azolic compounds has increased in recent years due to point mutations or upregulation of the *ERG11* gene (or Cyp51 enzyme) as well as in other genes of the ergosterol pathway (*ERG3*) or genes involved in the drug efflux pumps (*CDR1*, *CDR2*, and *MDR1*) that allow efficient exclusion of internal antifungal drugs [15]. These types of resistance mechanisms have been identified in the four priority fungal pathogens. The presence of active biofilms or alterations in metabolic pathways leading to a reduction or loss of function are also additional ways to acquire resistance to azoles in *C. albicans* and other fungi [15].

### 1.3. Echinocandins

These semisynthetic lipopeptides represent the most recently approved family of antifungals (first decade of the 21st century) that target the fungal cell wall. Three compounds are in clinical use: caspofungin (CAS), micafungin (MCF), and anidulafungin, but a fourth echinocandin, rezafungin, has recently been approved [25]. Echinocandins act as non-competitive inhibitors of the β-(1,3)-D-glucan synthase that catalyzes the formation of β-glucan polymers, impeding the biosynthesis of essential components of the fungal cell wall. The main therapeutic limitation of echinocandins is their restricted and rather diverse range of antifungal activity. Thus, they possess a strong fungicidal action against clinical isolates of *Candida* spp., while their effect on *Aspergillus* spp. is mainly fungistatic, and most pathogenic *Cryptococcus* spp. are refractory to echinocandins treatment, including rezafungin [25]. Moreover, a growing number of opportunistic yeasts have acquired resistance to echinocandins due to mutations in genes that encode the glucan synthase complex, which is essential for cell wall architecture [26,27]. Likewise, some side-off gastrointestinal and cardiovascular effects have been reported in treated patients [16].

## 2. The Search for New Antifungal Targets Is a Therapeutic Need

The search for new antifungal drugs is an urgent need due to the increasing incidence of systemic fungal infections and the emergence of antifungal-resistant pathogens that cannot be counteracted with the current clinical therapies [28]. Different compounds, directed mainly to invasive mycoses, are under clinical trials [29]. The emerging antifungals display diverse mechanisms of action and interact with different components. Compounds acting on traditional targets such as cell wall polymer biosynthesis, specific plasma membrane elements (e.g., ergosterol), or intracellular metabolism are currently under study. Alternative strategies, such as biofilm or mycelium development and adjuvant immunotherapy, are also being investigated (see [29] for more details). In fact, rezafungin and fosravuconazole (for the treatment of mycetoma) have been approved in recent years [25,30,31].

The antifungals in clinical use are directed against specific metabolic or biosynthetic fungal pathways of fungi. Nevertheless, a major challenge in antifungal chemotherapy is the eukaryotic nature of fungi. Since infectious pathogens and their hosts share similar cellular structures, this results in low selective toxicity for treatments. Therefore, exploring alternative antifungal targets could lead to new therapeutic strategies. One promising target unique to fungi is the two-component system, which is absent in animal cells [32]. These signaling systems are positioned upstream of Stress-Activated Protein Kinase (SAPK) pathways, essential for proper adaptation to environmental stresses.

## 3. Sensing Antifungals by SAPK Pathways in WHO High-Priority Fungal Pathogens

**Figure 1:**
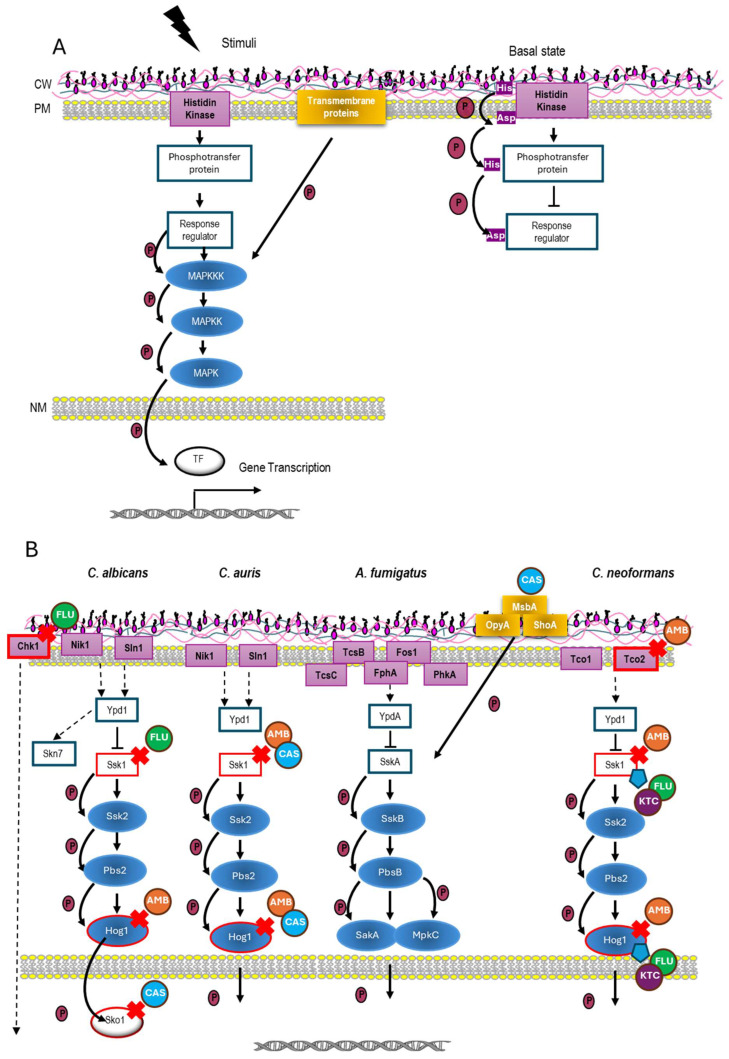
SAPK pathway in fungi. (**A**) Schematic representation of a SAPK signaling cascade. The pathway is depicted using generic nomenclature and the conserved MAP kinase module is depicted as blue ellipses The multistep phosphorylation mechanism in the two-component system in the absence of a stimulus is depicted on the right, while in the presence of stimuli, two-component phosphorylation is prevented, triggering the MAPK module phosphorylation (on the left). A secondary signaling branch identified in *S. cerevisiae* and *A. fumigatus* is also shown (yellow square). This signaling is mediated by different transmembrane proteins such as Msb2, Sho1, and Opy2. (**B**) Schematic representation of the molecular components of the SAPK signaling pathway in *C. albicans*, *C. auris*, *A. fumigatus*, and *C. neoformans*. Red crosses and blue shields tag those components of the pathways whose genetic deletion increases susceptibility (red crosses) or resistance (blue shields) to the indicated antifungal drugs depicted beside them.

MAPK (Mitogen-Activated Protein Kinase)-mediated pathways are signaling cascades widely conserved in all eukaryotic organisms, playing essential functions in cell physiology, such as mating, cell integrity, vegetative growth, and stress response [33,34,35]. In particular, they appear to perform a main role in the pathobiology of fungi [36,37]. The general structure of a prototypic MAP kinase consists of a module of three MAP kinases, which activate each other through phosphorylation (Figure 1A). This module receives signals through different mechanisms, such as transmembrane sensors coupled to GTPase proteins, two-component systems, other protein kinases, or intracellular sensors [37]. Although the MAPK module is strictly conserved, differences have been reported in upstream and downstream elements, as well as crosstalk and regulation among fungi (Figure 1).

Within the large group of MAP kinases, a subfamily present only in animals and fungi has recently emerged. This subfamily, named “Stress-activated protein kinase” (SAPK), is crucial in fungi for adaptation to acute environmental stimuli, including the sensing and subsequent signal transduction of antimycotic agents [38]. These SAPKs have been broadly studied in the yeast models *Saccharomyces cerevisiae* and *Schizosaccharomyces pombe* and, to a lesser extent, in the pathogenic fungi *C. albicans*, *C. auris*, *A. fumigatus*, and *C. neoformans* [37]. In some fungal species, the SAPK pathway has been termed the High-Osmolarity Glycerol response (HOG) pathway, and consequently, the MAPK of the pathway was named Hog1While the MAPK modules of *S. cerevisiae* and *A. fumigatus* can be activated through two independent branches (Figure 1A), only a single signaling branch triggers the Hog1 MAPK counterparts in the remaining priority fungal pathogens [37].

Interestingly, this conserved signaling mechanism is based on a two-component system. These systems are common parts of signal transduction pathways, widely found in prokaryotes, plants, and fungi, but they seem to be absent in animal cells [34,35]. In most eukaryotes, the two-component system functions as a multi-step phosphate transfer system involving four sequential phosphorylation events (Figure 1A). This phosphorelay system is initiated with the autophosphorylation of a histidine residue in the histidine kinase (sensor). Then, phosphate is sequentially transferred to an acid aspartic residue also in the Histidine Kinase, to be later transferred to a histidine residue in an intermediate phosphorelay (phosphotransfer) protein. Finally, phosphate is transferred to an acid aspartic residue in a response regulator protein [39]. In the presence of stimuli, initial autophosphorylation is prevented. Dephosphorylation of the response regulator protein is a potent signal to activate the MAPK module [36,39]. The role of SAPK pathways in cellular responses to various environmental stresses has been well documented [37,38,39,40]. Notably, among these stresses, the HOG pathway homologs can detect and respond to several antifungal agents. In fact, mutants lacking these signaling pathways are more vulnerable to certain antifungals. In this review, we highlight the structural similarities and differences among SAPK pathways in high-priority fungal pathogens, emphasizing their role in detecting antifungal compounds.

### 3.1. SAPK Pathway in C. albicans: The HOG Pathway

*C. albicans* remains the most common fungal pathogen in humans worldwide, where it tends to colonize the oral cavity, gut, and vagina. Productive infections occur preferentially when immune defenses are compromised, leading to candidiasis that can range from superficial to systemic [40]. The HOG pathway in *C. albicans* encompasses a two-component system and the MAPK module, which is composed of the proteins Ssk2 (MAPKKK), Pbs2 (MAPKK), and Hog1 (MAPK) (Figure 1B). This module is activated by a two-component system in which are involved three histidine kinase sensors, termed Sln1, Nik1, and Chk1, whose specific function remains unclear, although Sln1 seems to play a predominant role in Hog1 signaling [41,42,43]. Extensive research has demonstrated the essential role of the HOG pathway in virulence and cellular response to several environmental stresses, including antimicrobial peptides [44,45,46]. Notably, deletion of the intermediate phosphorelay protein Ypd1 in *C. albicans* enhances the degree of virulence [47], compromising its use as an antifungal target.

Interestingly, the HOG pathway is activated in response to AMB, whereas a set of *hog1Δ* null mutants display hypersensitivity to this polyene [48]. Hog1 phosphorylation, together with its intrinsic kinase activity, is required to deal with AMB exposure in *C. albicans* [48]. In the case of echinocandins, a conspicuous CAS-induced Hog1-activation was recorded with simultaneous stimulation of the oxidative stress response [49], whereas Hog1p is not phosphorylated after MCF supply [50,51]. Notably, the sensing and further response to CAS is partially dependent on the transcription factor Sko1 in a HOG-independent way [52]. Moreover, *ssk1* and *chk1* mutants are highly sensitive to FLC and VRC [53]. In summary, although *C. albicans* can respond to antifungals through distinct alternative mechanisms (e.g., the transcription factor Sko1), the impairment of the HOG pathway at different levels leads to a higher susceptibility to antifungals commonly used to treat candidiasis. Thus, this scenario suggests that it might be worthwhile to explore new formulations of combinatory therapy.

### 3.2. SAPK Pathway in Candidozyma auris

Since the first report in Japan in 2009, *C. auris* has spread rapidly and is widely distributed, establishing itself as a universal multidrug-resistant fungus. In fact, clinical strains refractory to azoles, echinocandins, and AMB, are frequently isolated in healthcare settings [6,7]. *C. auris* can persist outside the host in fomites, such as plastic surfaces, for at least 14 days, which favors nosocomial infections [54]. In turn, it also remains active on the skin for prolonged periods, contributing to inter- and intra-hospital transmission. A comparison between *C. auris* and other *Candida* species, such as *C. albicans*, revealed that *C. auris* is more resistant to hydrogen peroxide, osmotic stress, cationic, and cell wall disrupting agents. In contrast, it is highly susceptible to organic acids and unable to grow in anaerobic environments [55].

The Hog1 MAPK homologs in *C. auris* (Figure 1B) play a key role in resistance to different stresses, but also in virulence, cell wall biogenesis, biofilm formation, and survival to macrophages and neutrophils ex vivo [56]. Regarding antifungal sensing, *C. auris* shows a similar behavior to that recorded in *C. albicans*. Thus, Ssk1 mediates the phosphorylation of Hog1 after exposure to CAS and AMB in *C. auris.* Likewise, *ssk1Δ* and *hog1Δ* null mutants are very susceptible to those compounds compared to the parental strains. However, differences in the roles played by Ssk1 and Hog1 in response to antifungal drugs have been reported among distinct clinical isolates [57]. This suggests that MAPK signaling transduction in *C. auris* is rather complex and depends on the specific clade analyzed [57]. Whatever the case, sensitivity to both AMB and CAS can be restored after impairing Ssk1 or Hog1 activities in *C. auris* [57]. Thus, therapies combining an HOG pathway inhibitor with clinical antifungals (e.g., AMB or CAS) could be a suitable alternative for combating multi-resistant *C. auris* strains.

### 3.3. SAPK Pathway in Aspergillus fumigatus: The SakA and MpkC Mediated Pathway

Among the numerous *Aspergillus* species, *A. fumigatus* is the most virulent in humans [3]. This fungus causes allergies and opportunistic infections with a high incidence in immunocompromised individuals, mainly in neutropenic patients. The growing isolation of *A. fumigatus* strains resistant to azoles and echinocandins is considered a potential public health concern [58,59].

Unlike other fungi, the HOG homolog in *A. fumigatus* is more complex. This SAPK pathway is integrated by a MAPKKK (SskB), a MAPKK, (PbsB), and two MAPKs: SakA and MpkC. These MAPKs share each other 68.4% identity but differ in their kinetic and physiological functions (Figure 1B). SakA mediates adaptation to antifungals and cold stress, while MpkC is involved in the use of carbon sources (reviewed by Day [37]). Both SakA and MpkC are relevant for virulence [60]. This pathway is also involved in the response to osmotic and oxidative stress, cell wall-damaging agents, and antifungals such as CAS and nikkomycin Z [60].

Two branches placed upstream mediate signaling to the MAPK module. The first consists of a fairly elaborated two-component system, since at least 4 histidine kinases have been reported to control the specific signaling through this pathway (see [37,61]) (Figure 1B). TcsB (later named SlnA) seems to be homologous to *C. albicans* Sln1, and the main sensor of this branch. Histidine kinases can phosphorylate the phosphorelay protein homolog to Ypd1, known as YpdA, which in turn phosphorylates the response regulator SskA [62].

A second signaling branch mediates the activation of both the HOG and the cell wall integrity pathway. This branch is composed of ShoA (Sho1 homolog), MsbA (Msb2 homolog), and OpyA (Opy2 homolog), and acts together with the two-component system in the full activation of SakA and MpkC in response to different stresses [62,63]. Among these, both SakA and MpkC are phosphorylated in response to CAS exposure [62]. This activation was significantly impaired in an *msbA* mutant, suggesting that the second branch plays a predominant role after CAS addition. Proteomic analyses in response to CAS treatments showed that PKA, CWI, and HOG pathways are closely coordinated [64]. Furthermore, transcriptomic arrays using clinical isolates of *A. fumigatus* indicated that exposure to ITC induces a set of transcriptional changes that involve, among others, some proteins belonging to the HOG pathway [65], suggesting that this pathway may be activated in the presence of this antifungal. Although in *A. fumigatus* the HOG homologous pathway involves multiple signaling branches, data suggest that preventing its activation increases the susceptibility to antifungals such as CAS [63]. Invasive aspergillosis is a severe disease that requires the use of agents such as VRC and AMB, although therapies using PSZ, ITC, CAS, and other echinocandins are also effective [11].

### 3.4. SAPK Pathway in Cryptococcus neoformans: The HOG Pathway

*C. neoformans* ranks first in the WHO list of high-priority fungal pathogens, since it causes fatal meningoencephalitis, mainly in immunocompromised individuals. A relevant attribute of its virulence is the presence of an antiphagocytic polysaccharidic capsule [3].

In *C. neoformans*, the HOG pathway controls mating, melanin and capsule production, as well as ergosterol biosynthesis and cellular ergosterol content [66]. Unlike other fungi, in a fraction of clinical isolates, Hog1 is constitutively phosphorylated under standard conditions and becomes actively dephosphorylated in response to different kinds of stress [67]. Activation of the MAPK module is carried out by a two-component system located upstream. This system is integrated by a response regulator, Ssk1, a phosphotransfer protein Ypd1, and seven histidine kinases, Tco1 to Tco7 [68] (Figure 1B). Tco1 and Tco2 have priority and specific functions, which somehow overlap, with Tco1 being more relevant for virulence in a murine model of meningitis [68] while Tco2 mediates susceptibility to AMB. In fact, mutants defective in Tco2 display sensitivity to AMB [66].

Transcriptomic analyses revealed that in *C. neoformans*, genes involved in sterol biosynthesis were upregulated in *hog1Δ* and *ssk1Δ* mutants, resulting in enhanced ergosterol content and hypersensitivity to AMB [66]. The analysis of strains with different levels of constitutive Hog1 phosphorylation showed a Hog1-induced repression of ergosterol biosynthesis under standard growth conditions. In contrast, *ssk1Δ* and *hog1Δ* mutants displayed increased resistance to KTC (imidazole) and FLC but not to ITC (triazole) [66]. These differential susceptibilities are consistent with the mechanisms of action of AMB and azoles. While AMB binds directly to ergosterol, forming pores and disrupting the membrane integrity, azoles inhibit Cyp51A, thereby reducing ergosterol synthesis. Therefore, higher ergosterol levels potentiate AMB toxicity by increasing target availability [22,69], whereas they attenuate azole efficiency by partially compensating for the enzymatic blockade induced by these drugs [70]. Furthermore, differences between FLC, FTC, and ITC may be attributable to species-specific differences in Erg11 protein sequence. For example, *A. fumigatus* Cyp51A enzyme (Erg11 ortholog) contains an isoleucine at position 301, where *C. albicans* has a threonine (T315). This single amino acid largely reduces FLC and KTC binding, explaining *A. fumigatus’s* intrinsic resistance to those drugs. ITC by contrast, retains activity due to its bulkier structure and stronger binding affinity, which are less affected by this variation [71]. Likewise, in *C. neoformans*, a Y145F substitution in Erg11 abolishes FLC and VRC activity, but paradoxically enhances susceptibility to ITC and PSZ, highlighting the existence of drug-specific interactions within the binding pocket [72].

In summary, inactivation of the HOG pathway increases both the biosynthesis and content of ergosterol, which confers sensitivity to AMB and resistance to FLC and KTC. The treatment of disseminated cryptococcal disease with risk of CNS perturbations is divided into three phases. In the first phase, a combination of AMB plus flucytosine is applied. The subsequent consolidation, maintenance, and prophylaxis phases are based on FLC, although other azoles can be used [73]. When implementing a combination therapy that includes AMB plus a potential HK inhibitor, the use of FLC should be avoided in favor of ITC to prevent treatment failure.

## 4. Histidine Kinase Inhibitors Under Study

Elements of the two-component system, either the histidine kinase or the response regulator, have long been considered appropriate antimicrobial targets [74,75]. Several molecules have been identified as potential histidine kinase/two-component system inhibitors [76], some of which are currently under investigation in preclinical phases (e.g., waldiomycin, walkmycin, closantel, among others) to treat different bacterial infections.

Several screening methods for identifying inhibitors of fungal histidine kinases have been developed, and their effectiveness has been tested with known histidine kinase inhibitors such as fludioxonil [64]. To date, none have been approved for human use, although fludioxonil is used in agriculture. Interestingly, this compound has been reported to activate the MAPK Hog1 in *C. neoformans*, while the *hog1* mutant was resistant to it [60]. To our knowledge, susceptibility of this fungus to treatment with fludioxonil combined with any clinical antifungal has not been reported. Additionally, it should be noted that fludioxonil itself may induce resistance to FLC in *C. albicans* [64]. This suggests that other histidine kinase inhibitors could cause a similar resistant phenotype in this or other fungi. This undesirable effect could be mitigated by combining this compound with other approved standard antifungals.

## 5. Conclusions and Perspectives

Current antifungal treatments are insufficient to address the growing issue of systemic mycosis caused by highly infectious fungi in humans. Therefore, alternative therapeutic strategies need to be explored. Here, we suggest targeting upstream elements of highly conserved SAPK pathways as potential antifungal options, due to their vital role in fungal biology, including virulence, cell morphogenesis, cell wall structure, and environmental stress responses. Initial data show that several antifungals can activate SAPK signaling, triggering a response aimed at fungal survival. Combining well-established antifungal drugs with formulations that prevent SAPK activation could enhance treatment effectiveness and reduce resistance development. However, the existing evidence is limited, and more robust data from both in vitro and in vivo studies are necessary.

## Data Availability

No new data were created or analyzed in this study.

## Abbreviations

WHO: World Health Organization; AMB: Amphotericin B; CAS: Caspofungin; MCF: Micafungin; ITC: Itraconazole; FLC: Fluconazole; KTC: Ketoconazole; VRC: Voriconazole; PSZ, Posaconazol; CNS: Central nervous system. HOG: High Osmotic Glycerol; MAPK: Mitogen Activating Protein Kinase; PKA: Protein Kinase A; CWI: Cell Wall Integrity; SAPK: Stress-Activated Protein Kinases.
